# Lifetime Exposure to a Constant Environment Amplifies the Impact of a Fructose-Rich Diet on Glucose Homeostasis during Pregnancy

**DOI:** 10.3390/nu9040327

**Published:** 2017-03-25

**Authors:** Aleida Song, Stuart Astbury, Abha Hoedl, Brent Nielsen, Michael E. Symonds, Rhonda C. Bell

**Affiliations:** 1 Division of Human Nutrition, Department of Agricultural, Food and Nutritional Sciences, University of Alberta, Edmonton, AB T6G 2E1, Canada; aleida@ualberta.ca (A.S.); stuart.astbury@nottingham.ac.uk (S.A.); abha@ualberta.ca (A.H.); banielse@ualberta.ca (B.N.); 2 Early Life Research Group, Academic Division of Child Health, Obstetrics & Gynaecology, and NIHR Nottingham Digestive Diseases Biomedical Research Unit, School of Medicine, University of Nottingham and Nottingham University Hospitals NHS Trust, Nottingham NG7 2UH, UK; 3 Women and Children’s Health Research Institute, University of Alberta, Edmonton, AB T6G 2E1, Canada

**Keywords:** fructose, development, pregnancy, metabolism, type II diabetes

## Abstract

The need to refine rodent models of human-related disease is now being recognized, in particular the rearing environment that can profoundly modulate metabolic regulation. Most studies on pregnancy and fetal development purchase and transport young females into the research facility, which after a short period of acclimation are investigated (Gen0). We demonstrate that female offspring (Gen1) show an exaggerated hyperinsulinemic response to pregnancy when fed a standard diet and with high fructose intake, which continues throughout pregnancy. Markers of maternal hepatic metabolism were differentially influenced, as the gene expression of acetyl-CoA-carboxylase was raised in Gen1 given fructose and controls, whereas glucose transporter 5 and fatty acid synthase expression were only raised with fructose. Gen1 rats weighed more than Gen0 throughout the study, although fructose feeding raised the percent body fat but not body weight. We show that long-term habituation to the living environment has a profound impact on the animal’s metabolic responses to nutritional intervention and pregnancy. This has important implications for interpreting many studies investigating the influence of maternal consumption of fructose on pregnancy outcomes and offspring to date.

## 1. Introduction

Diabetes and metabolic syndrome are normally considered to result from exposure to an obesogenic environment together with an individual’s genotype [1]. It is also recognized that in populations that migrate to more affluent countries, it is the next generation that is at greater risk of becoming diabetic, in part due to early life exposures [2,3]. These factors are seldom considered in animal models of diabetes, especially those examining the impact of exposure to an adverse maternal nutritional environment on the offspring. Although there is increasing awareness of the role of high intakes of fructose in metabolic syndrome–associated diseases, such as non-alcoholic fatty liver disease [4], coronary artery disease [5] and type II diabetes [6], and a growing body of literature covering high intakes of fructose during pregnancy [7,8,9,10], there is still a lack of longer-term rodent studies following the offspring into adulthood and pregnancy. Rodent studies have demonstrated that fructose in pregnancy can significantly reduce the weight of the placenta [11], and increase the expression of genes related to lipogenesis in the offspring liver [12].

Rodent models of nutritional programming usually use young females that are shipped into a research facility and, after a brief period of acclimation, are exposed to a dietary intervention. This experimental approach seldom accounts for the fact that the mothers experienced a significant change in environment while offspring were not moved from their home environment. Failure to account for these differences could mask important adverse consequences that are expressed only in subsequent generations that are fully habituated to their living environment. This could account for how diet-induced maternal obesity can result in glucose intolerance in the offspring in some [13,14] but not all studies [15,16]. We therefore examined whether offspring born and reared in a single research facility (Gen1) exhibit a more pronounced diabetes-related phenotype than their mothers (Gen0). Half of the animals were given fructose (F) in drinking water as a 10% solution (vs. distilled water) prior to and during pregnancy to establish whether fructose would amplify these effects. Fructose was chosen since it produces a gestational diabetes mellitus (GDM)-type phenotype of metabolic dysfunction in the adult offspring [8,11,17]. A 10% solution was chosen to more closely mimic fructose consumption in humans (i.e., as an added sweetener), and previous work has shown this concentration has significant metabolic effects in rodents [18]. The extent to which metabolic outcomes differ between generations has not been previously examined.

## 2. Materials and Methods

### 2.1. Animals and Diets

Seven-week-old female Wistar rats (Charles River Canada, Montreal, Quebec, Canada) were pair-housed in shoebox cages in a temperature-controlled room (21–23 °C; 40%–70% humidity) with a 12 h light:dark cycle. All rats were allowed access to food ad libitum (Purina 5001; Purina Mills, St. Louis, MO, USA) throughout the study and distilled water was available to all rats during the standard one week acclimation period. At eight weeks of age, rats were randomly assigned to receive either a 10% fructose solution (*w*/*v* in distilled water, Amresco, Solon, OH, USA; Gen0-F, *n* = 15) or distilled water (Gen0-C, *n* = 15). At 11 weeks of age, they were co-housed with males which had been maintained on distilled water. Pregnancy was confirmed by vaginal lavage and a positive sperm test was considered gestational day (GD) 0. Animals remained on this intervention for three weeks prior to mating and during mating and pregnancy. Ten Gen0-C and 10 Gen0-F were euthanized at GD 21 (details described below). The remaining pregnant dams were left to litter out and all litters were culled to 10–12 pups/litter at birth. Dams continued their assigned diet during lactation (until 21 days after delivery), at which point two female pups were randomly selected to remain in the study as offspring. Eight-week-old female offspring were placed on the same diet as their dams (either 10% fructose solution (Gen1-F, *n* = 10) or distilled water (Gen1-C, *n* = 10). These diets continued through mating and pregnancy. Pregnant offspring were euthanized at GD 21. The study was approved by the Research Ethics Office of the University of Alberta.

### 2.2. Regular Monitoring of Body Weight and Plasma Metabolites

Body weights were recorded weekly during the study, beginning at eight weeks of age and in early (GD4-7), mid (GD14-17) and late pregnancy (GD19-20). Morning blood samples were also collected, mixed with anti-coagulant (K_2_ EDTA, BD, Franklin Lakes, NJ, USA) and remained on ice until centrifugation (Eppendorf Centrifuge 5415C, Germany, 16,000× *g*, 5 min). Plasma was stored at −20 °C until being analyzed for glucose (Trinder assay kit, Genzyme Diagnostics, Charlottetown, PEI, Canada), insulin (Rat Ultrasensitive ELISA Immunoassay kit, ALPCO Diagnostics, Salem, NH, USA), and triglycerides (Triglyceride-SL assay kit, Genzyme Diagnostics, Cambridge, MA, USA).

### 2.3. Oral Glucose Tolerance Tests (OGTT)

OGTT were carried out on GD19 after a 4 h fast, this was chosen both to avoid inducing a starvation state overnight [19,20] and to avoid the stress fasting places on pregnant rodents due to their increased glucose utilization [21]. Following collection of a baseline blood sample a 3 g·kg^–1^ glucose solution was administered by gavage. Blood samples were collected at 15, 30, 45, 60 and 90 min after glucose administration. Plasma was separated and stored as above before assaying for glucose and insulin. The incremental area under the curve (IAUC) for glucose and insulin from 0 to 90 min was calculated.

### 2.4. Determination of Body Composition and Tissue Collection

On GD 21 the proportions of fat and lean tissue of each animal were measured using quantitative magnetic resonance imaging (EchoMRI LLC 4-in-1 whole-body composition analyzer; Echo Medical Systems, Houston, TX, USA). Following euthanasia, the liver was excised from each rat, snap-frozen in liquid nitrogen and stored at −80 °C until analysis. Placentae and fetuses were dissected from the uterus, counted, individually weighed, and the placental:fetal weight ratio calculated.

### 2.5. RNA Extraction and Determination of Hepatic Gene Expression

Total RNA was extracted from frozen liver that had been homogenized in TRI Reagent (Ambion Diagnostics, Austin, TX, USA), using the RNeasy Mini Kit (Qiagen N.V., Hilden, Germany). RNA concentration and purity were confirmed using a Nanodrop spectrophotometer (Thermo Scientific, Waltham, MA, USA), and reverse transcription PCR was carried out using the High Capacity cDNA reverse transcription kit (Applied Biosystems, Waltham, MA, USA). Quantitative polymerase chain reaction (qPCR) was carried out using SYBR Green dye and the StepOne Plus PCR machine (Applied Biosystems). Primers were designed using Primer3 [22] to the rat genome; primer sequences and GenBank references are included in Supplementary Table S1. Primers were designed to be intron-spanning to avoid amplification of genomic DNA. Product sizes and primer specificity were confirmed using classical PCR and gel electrophoresis before qPCR and melt-curves following qPCR. All qPCR results were adjusted to two reference genes (RPLP0 and GAPDH) using GeNorm for Microsoft Excel [23] and are presented as fold change in arbitrary units relative to the Gen0-C group, according to the 2^−ΔΔCT^ method [24].

### 2.6. Statistical Analysis

Following a Shapiro Wilk test for normality all data were compared using unpaired *t*-tests, with a Bonferroni correction applied where necessary for multiple comparisons. Analyses were carried out using SPSS (version 23, IMB Corp., Armonk, NY, USA).

## 3. Results and Discussion

**Figure 1:**
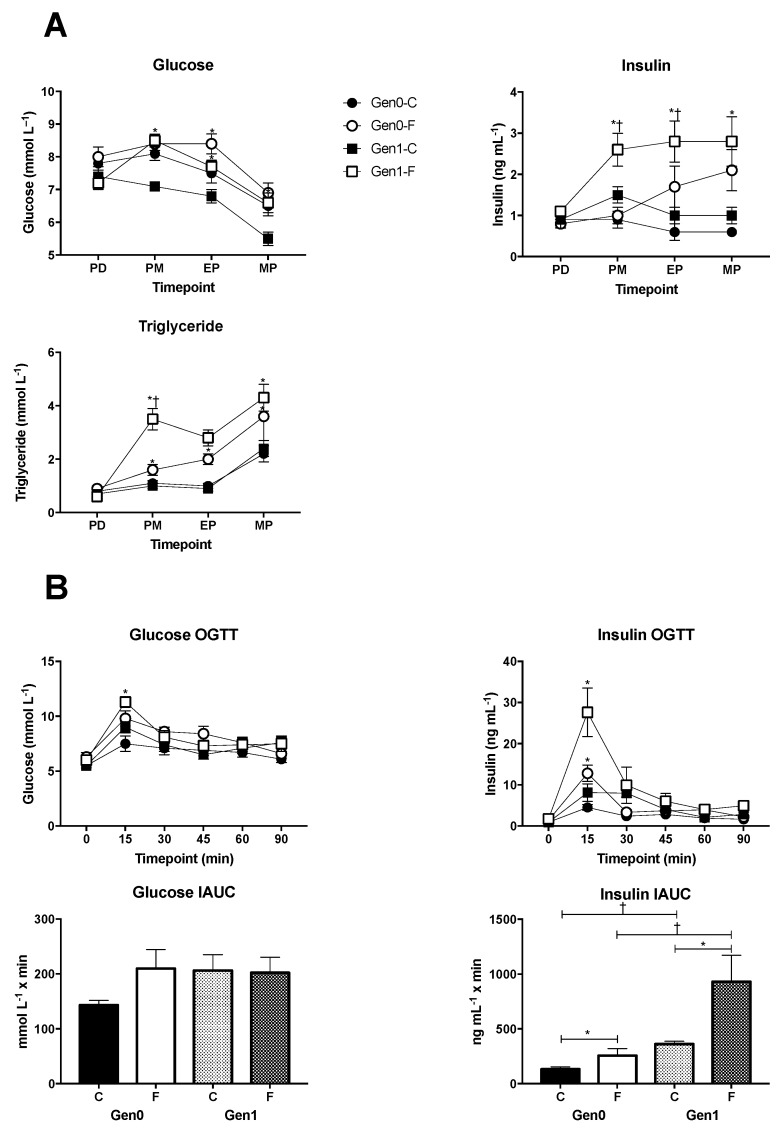
(**A**) Plasma glucose, insulin and triglyceride concentrations measured regularly throughout the study. Gen0-C *n* = 15, Gen0-F *n* = 15, Gen1-C *n* = 10, Gen1-F *n* = 10. Time points were as follows: PD/pre-diet treatment = eight weeks of age, PM/pre-mating = 11 weeks, EP/early pregnancy = 12 weeks/gestational day (GD) 4–7, MP/mid pregnancy = 13 weeks/GD14–17. Whole blood was collected from non-fasted rats and plasma was separated and stored at −20 °C before analysis. (**B**) Oral glucose tolerance tests (OGTT) were conducted on GD19. Rats were fasted for 4 h. Following collection of a baseline blood sample 3 g·kg^–1^ body weight of glucose was administered by gavage. Blood samples were collected at 15, 30, 45, 60 and 90 min post glucose bolus. * *p* < 0.05, Gen0-C vs. Gen0-F, or Gen1-C vs. Gen1-F (effect of diet) and † *p* < 0.05, Gen0-C vs. Gen1-C and Gen0-F vs. Gen1-F (effect of generation), unpaired *t*-test with a Bonferroni correction applied for multiple comparisons.

Fructose feeding caused hyperinsulinemia to a greater extent in Gen1 than in Gen0 (Figure 1A). Hyperinsulinemia began prior to pregnancy, was exacerbated during pregnancy, and was also observed during the OGTT at the end of pregnancy (Figure 1B). This suggests increased insulin resistance in Gen1 that is exacerbated by a high intake of fructose and was corroborated by the fact that the insulin IAUC was greater in Gen1 than in Gen0 and exaggerated in Gen1-F. Consistent with evidence in humans and rats [25,26], circulating triglycerides were higher in Gen0-F and Gen1-F prior to and during pregnancy, and more so in Gen1.

Body weight was raised in Gen1, but composition did not differ between generations. Fructose enhanced the proportion of fat in Gen0 and Gen1 (Table 1). Litter size was not different between generations or diet groups but fetal weight was reduced in Gen0-F and Gen1-F, and placentae were smaller in Gen1-F (Table 1). Reductions in placental but not fetal weight following fructose intake have been previously demonstrated [11]. Further investigation into the generational effects of fructose on placental blood flow and nutrient transport would be worthwhile.

**Figure 2:**
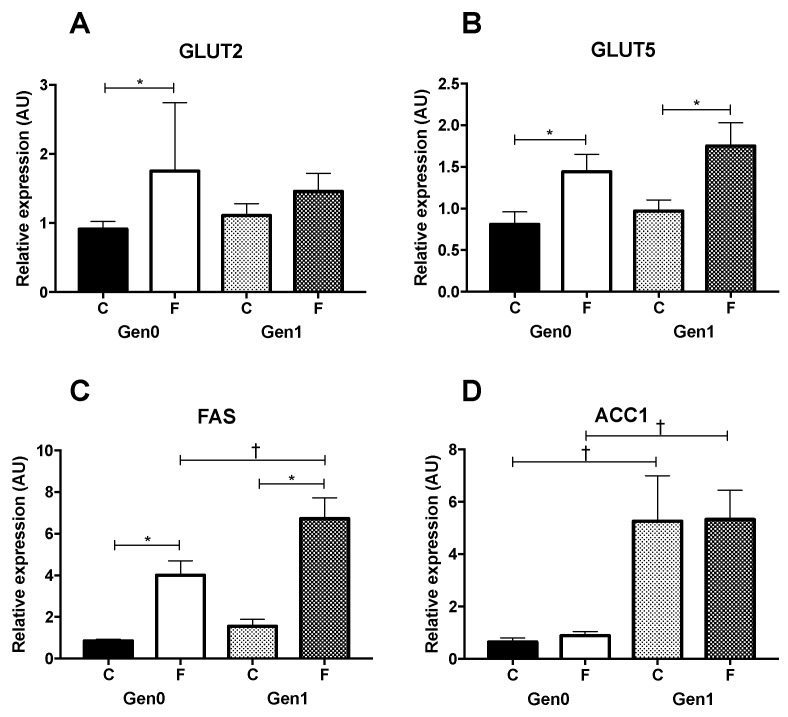
Hepatic gene expression. Gen0-C *n* = 10, Gen0-F *n* = 10, Gen1-C *n* = 10, Gen1-F *n* = 10. Livers were excised on gestational day 21 and snap frozen before RNA extraction and cDNA production by reverse transcription PCR. Expression of glucose transporter 2 (*GLUT2*) (**A**), fructose transporter *GLUT5* (**B**), fatty acid synthase (*FAS*) (**C**) and acetyl-CoA-carboxylase (*ACC1*) (**D**) were measured by real-time PCR, relative to the reference genes glyceraldehyde-3-phosphate dehydrogenase (*GAPDH*) and 60 s acidic ribosomal protein P0 (*RPLP0*) using GeNorm [15] and the 2^−ΔΔCT^ method [16]. * *p* < 0.05, Gen0-C vs. Gen0-F, or Gen1-C vs. Gen1-F (effect of diet) and † *p* < 0.05, Gen0-C vs. Gen1-C and Gen0-F vs. Gen1-F (effect of generation), unpaired *t*-test with a Bonferroni correction applied for multiple comparisons.

Given the importance of the liver in responding to high fructose intake [27], we examined the mRNA abundance of genes involved in liver glucose and fructose transport [28,29]. Though *GLUT2* expression was raised by fructose in Gen0 and *GLUT5* was raised in Gen0 and Gen1, neither was affected by generation (Figure 2). Generational effects were observed in *FAS* and *ACC1* expression, and *FAS* expression also displayed a fructose effect. This suggests an upregulation in lipid metabolism in Gen1 that is more pronounced with fructose intake.

The substantial difference in insulin resistance and hypertriglyceridemia through pregnancy between Gen0 dams that were recently transported to the facility and Gen1 dams that were born and maintained in the same environment for their life has not been previously demonstrated. Moreover, the nutritional intervention negatively impacted insulin resistance and triglyceride concentrations before and through pregnancy, and these responses were enhanced in Gen1. This is consistent with previous work, which suggests that intake of fructose inversely correlates with insulin receptor expression in a number of organs [30]. To our knowledge, no previous studies have compared the insulin response to fructose in pregnancy between dams and their offspring. The fact that insulin responses were higher in both groups of offspring, who were heavier and fatter than their mothers, suggests that body weight and adiposity could play a role. Previous reports on fructose consumption leading to hyperleptinemia support this [27,31]. The amplified insulin response to glucose in Gen1 has clear implications for the interpretation of studies previously conducted examining the impact of maternal diet on long-term outcomes in offspring. It also highlights the importance of thoroughly characterizing both the mother and her offspring so that true programming effects can be identified. It is likely that in studies in which young rodents were shipped to a research facility and studied a few weeks later, animals were still adapting to this transition. Based on our findings, we suggest that the negative impact of nutritional programming on offspring glucose and lipid homeostasis mediated by fructose feeding [11,12,32] has been significantly underestimated.

A primary factor in enabling us to identify the profound effect on insulin and lipid profiles was our focus on pregnancy as a major physiological challenge. As expected, plasma insulin became raised during pregnancy, but this adaptation was greatly amplified by fructose exposure. At the start of the study (i.e., when the animals were eight weeks old), age-matched offspring were heavier than their dams, and interestingly, the increased Gen1 body weight was not accompanied with greater adiposity, although it was raised in both groups by fructose (Table 1). The impact of adding purified fructose to the diet in human beings remains controversial due in part to the inconsistency in metabolic outcomes [33]. Studying animals which have recently been subjected to the combined stress of transportation and a new living environment may mask many of the metabolic consequences of fructose.

## 4. Conclusions

In conclusion, substantial adaptations in metabolic profiles through pregnancy are seen between generations that are greatly amplified when offspring are maintained in the research environment as opposed to being transported into the facility and then studied. The magnitude of this adaptation is most apparent in the regulation of plasma insulin and lipids and is exacerbated when the animals are allowed ad libitum access to a fructose solution. It is therefore likely that many studies examining the impact of dietary modulation through pregnancy underestimate the magnitude of the effect on both the mother and her offspring.

## Figures and Tables

**Table 1:** Body weights (g) before and throughout pregnancy, and body composition and feto-placental unit near to term.

Age/Pregnancy Stage	Gen0-C	Gen0-F	Gen1-C	Gen1-F
Body Weight (g)				
Week 8/Pre-diet	200.6 ± 3.3	204.1 ± 3.1	225.1 ± 6.3 ^†^	226.4 ± 5.7 ^†^
Week 9	227.0 ± 2.0	238.3 ± 2.9	254.5 ± 6.6 ^†^	252.3 ± 6.5 ^†^
Week 10	247.3 ± 2.3	257.2 ± 3.1	280.7 ± 6.8 ^†^	285.9 ± 7.7 ^†^
Week 11/Pre-mating	261.5 ± 3.0	277.7 ± 4.7	305.5 ± 7.4 ^†^	316.3 ± 9.3 ^†^
Week 12/ Early pregnancy	290.5 ± 2.3	304.9 ± 5.3	332.2 ± 6.8 ^†^	352.3 ± 12.9 ^†^
Week 13/ Mid pregnancy	329.4 ± 5.1	337.6 ± 5.9	383.8 ± 7.6 ^†^	393.3 ± 14.3 ^†^
Week 14/ Late pregnancy	386.5 ± 5.4	403.1 ± 6.7	428.7 ± 12.3 ^†^	444.4 ± 15.6 ^†^
Body Composition (%)				
Fat mass at GD21	11.2 ± 0.7	15.2 ± 1.0 *	11.4 ± 0.5	16.7 ± 1.3 *
Feto-placental unit				
Number of pups	15.5 ± 1.7	16.0 ± 3.2	16.3 ± 2.3	17.0 ± 3.3
Placental weight (g)	0.53 ± 0.02	0.48 ± 0.02	0.57 ± 0.02	0.47 ± 0.01 *
Fetal weight (g)	3.88 ± 0.19	3.36 ± 0.14 *	4.07 ± 0.05	3.57 ± 0.09 *
The ratio of placental:fetal weight	0.14 ± 0.01	0.15 ± 0.01	0.14 ± 0.00	0.13 ± 0.01

All values are means ± SEM. Gen0-C *n* = 10, Gen0-F *n* = 10, Gen1-C *n* = 10, Gen1-F *n* = 10. * *p* < 0.05, Gen0-C vs. Gen0-F, or Gen1-C vs. Gen1-F (effect of diet) and † *p* < 0.05, Gen0-C vs. Gen1-C and Gen0-F vs. Gen1-F (effect of generation), unpaired *t*-test with a Bonferroni correction applied for multiple comparisons.

## Supplementary Materials

The following are available online at www.mdpi.com/1999-4907/9/04/327/s1, Table S1: Primer sequences.
