# Supplementary material for: Lifetime Exposure to a Constant Environment Amplifies the Impact of a Fructose-Rich Diet on Glucose Homeostasis during Pregnancy
Source: Nutrients. 2017 Mar 25;9(4):327. doi: 10.3390/nu9040327 (PMC5409666; doi:10.3390/nu9040327)
Supplement: Supplementary file 1 [file nutrients-09-00327-s001.docx]

**Table S1.** Primer sequences.

| **Gene** | **Primer Sequence** | **Accession (GenBank)** |
| --- | --- | --- |
| Glucose transport | | |
| Glucose transporter 2 (*GLUT2*) | Forward: AAAGCCCCAGATACCTTTACCT | NM_012879.2 |
|  | Reverse: TGCCCTTAGTCTTTTCAAGC |  |
| Glucose transporter 5 (*GLUT5*) | Forward: CTTCGGAGTGTCTTGGAAGC | NM_031741.1 |
|  | Reverse: GGCAGGGACTCCAGTCAG |  |
| Fat metabolism | | |
| Fatty acid synthase (*FAS*) | Forward: AAGCGGTCTGGAAAGCTGAA | NM_017332.1 |
|  | Reverse: ACCAGTGTTTGTTCCTCGGA |  |
| Acetyl-CoA carboxylase 1 (*ACC1*) | Forward: GTACAACGCAGGCATCAGAA | NM_022193.1 |
|  | Reverse: AGTCCCAGCGCTCACATAAC |  |
| Reference genes | | |
| Glyceraldehyde-3-phosphate dehydrogenase (*GAPDH*) | Forward: TGGAGTCTACTGGCGTCTT | XM_017593963.1 |
|  | Reverse: TGTCATATTTCTCGTGGTTCA |  |
| 60s acidic ribosomal protein P0 (*RPLP0*) | Forward: GATGCCCAGGGAAGACAG | NM_022402.2 |
|  | Reverse: CACAATGAAGCATTTTGGGTAG |  |
